# Supplementary material for: Explaining the apparent impenetrable barrier to ultra-relativistic electrons in the outer Van Allen belt
Source: Nat Commun. 2018 May 10;9:1844. doi: 10.1038/s41467-018-04162-3 (PMC5945634; doi:10.1038/s41467-018-04162-3)
Supplement: Supplementary file 1 — Supplementary Information [file 41467_2018_4162_MOESM1_ESM.pdf]

SUPPLEMENTARY INFORMATION

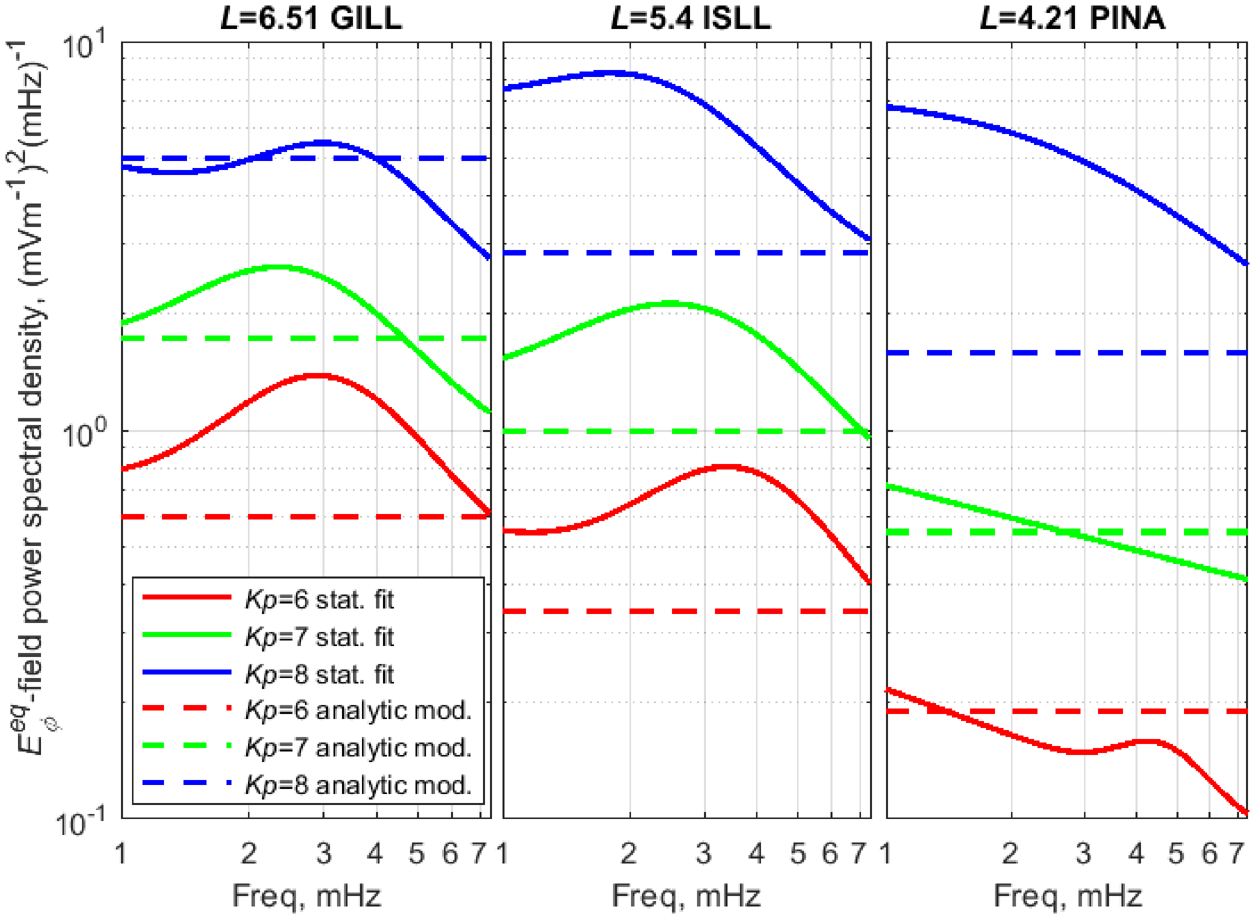

Supplementary Figure 1: Comparison between the Ozeke et al.[1] analytic model diffusion coefficients and those from statistical fits to a combination of a power law and a local Gaussian power peak from Ozeke et al.[2]. Solid lines show the azimuthal component of the electric field power spectral density derived from ground-based magnetometer data in the Ozeke et al.[1] analytic (dotted line) and Ozeke et al.[2] fitted power law models (solid line) for ground-magnetometer stations at Gillam (GILL), Island Lake (ISLL) and Pinawa (PINA) in the CARISMA magnetometer array (see Mann et al.[3] ; www.carisma.ca). Results are shown for *Kp* = 6, 7 and 8. The agreement between both models is very good, justifying the use of the Ozeke et al.[1] model even for the three 3-hour periods when *Kp*>6. Note also, however, that for *Kp* = 8 the actual statistical fitted ULF wave power (solid line) increases significantly above that of the analytic model (dotted line). See also the discussion related to Figure 4 in the main article for high *Kp*.

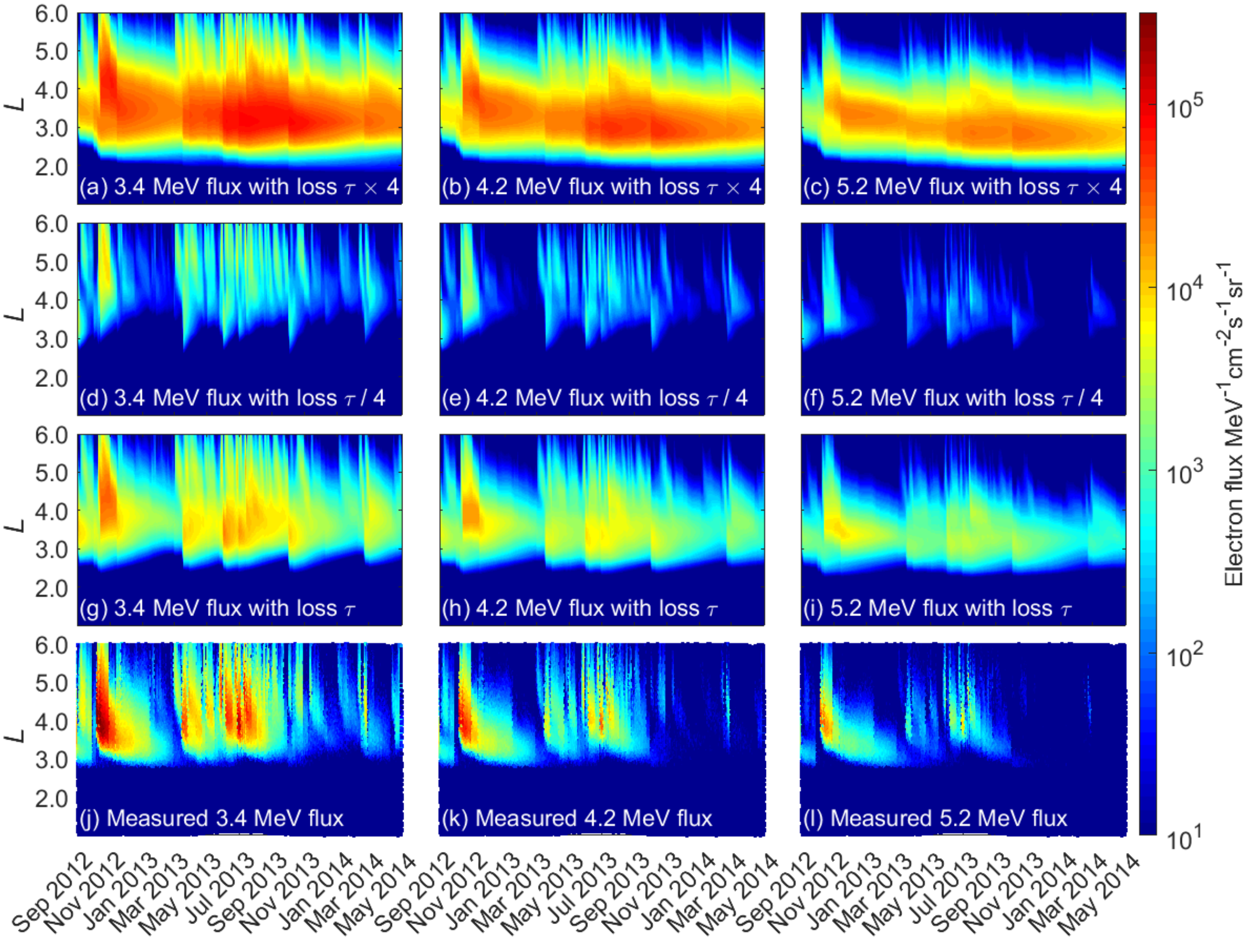

Supplementary Figure 2: The dependence of simulated electron flux profiles on the plasma wave-particle loss lifetime, τ. The simulated electron flux is derived using the radial diffusion coefficients from Ozeke et al.[1] and the electron loss lifetimes, τ, from Orlova et al.[4] and Gu et al.[5] inside and outside the Carpenter and Anderson [6] *Kp*-dependent empirical plasmapause. These analytic models for the electron loss lifetime, τ, can differ by a factor of 4 from the electron lifetimes derived directly from in-situ VLF wave measurements, see e.g., Orlova et al.[4]. Panels (a), (b), and (c) show the impact of increasing these electron loss lifetimes by a factor of 4, and panels (d), (e), and (f) show the impact of decreasing the electron lifetimes by a factor 4, as compared to simulations using the loss timescale, τ, shown in panels (g), (h) and (i). Van Allen Probe measured electron flux is shown in panels (j), (k), and (l) - lying somewhere between the simulation results using $\tau \times 4$ and $\tau/4$, and relatively close to the results using τ itself. Taking account of the uncertainties in the electron lifetime models, a better agreement might be able to be obtained between the simulated and observed electron fluxes at energies of at 3.4 MeV, 4.2 MeV and 5.2 MeV. Nonetheless, the feature of the apparent impenetrable barrier is seen in all simulations, showing that its location is not a sensitive function of the rate of loss. Instead the impenetrable barrier location is largely determined by ULF wave radial transport, including the effects of the finite duration of the driver and the steep L-shell gradient of ULF wave radial diffusion coefficient.

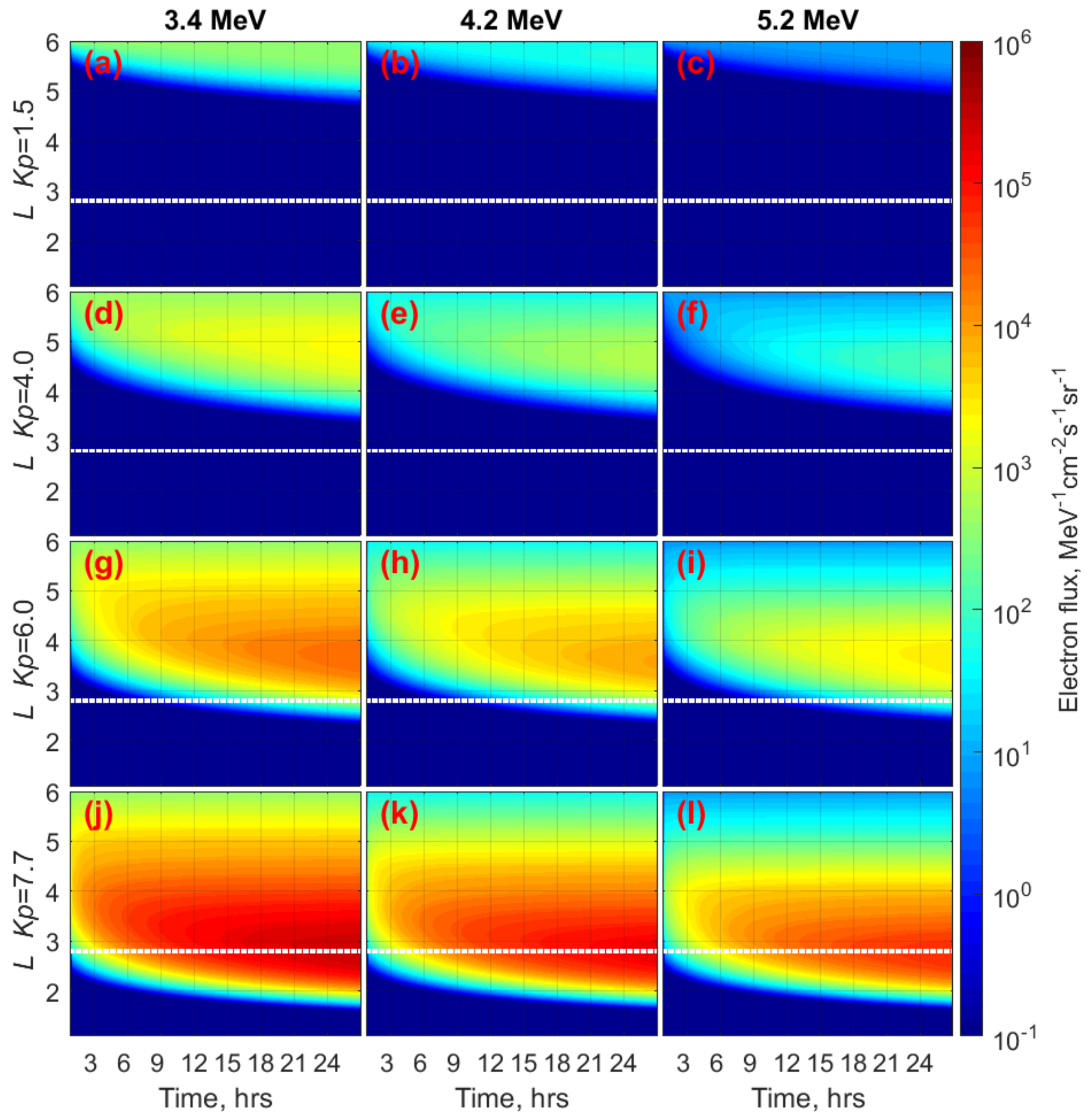

Supplementary Figure 3: Electron flux simulations over 26 hours driven by constant ULF wave radial diffusion coefficients defined by fixed *Kp*. Similar to Figure 3 in the main article, each column shows the resulting flux at fixed energies of 3.4 MeV (left), 4.2 MeV (middle) and 5.2 MeV (right) arising from transport at fixed *Kp* of 1.5 (top row), 4 (second row), 6 (third row), and 7.7 (bottom row) using D_{LL}[Ozeke]. The results are derived from a series of first adiabatic invariant conserving simulations in a dipole magnetic field. Here the results are plotted over a shorter time interval of 26 hours than in Fig. 3 of the main article so the time taken for the electron flux to diffusion inward below L=2.8 during for $Kp \gtrsim 6$ can be clearly identified. Panels (j), (k) and (l) show that after $\gtrsim$6 hours of inward radial diffusion specified by D_{LL}[Ozeke] with Kp=7.7 the ultra-relativistic electron flux can penetrate below L=2.8, indicated by the white dotted line.

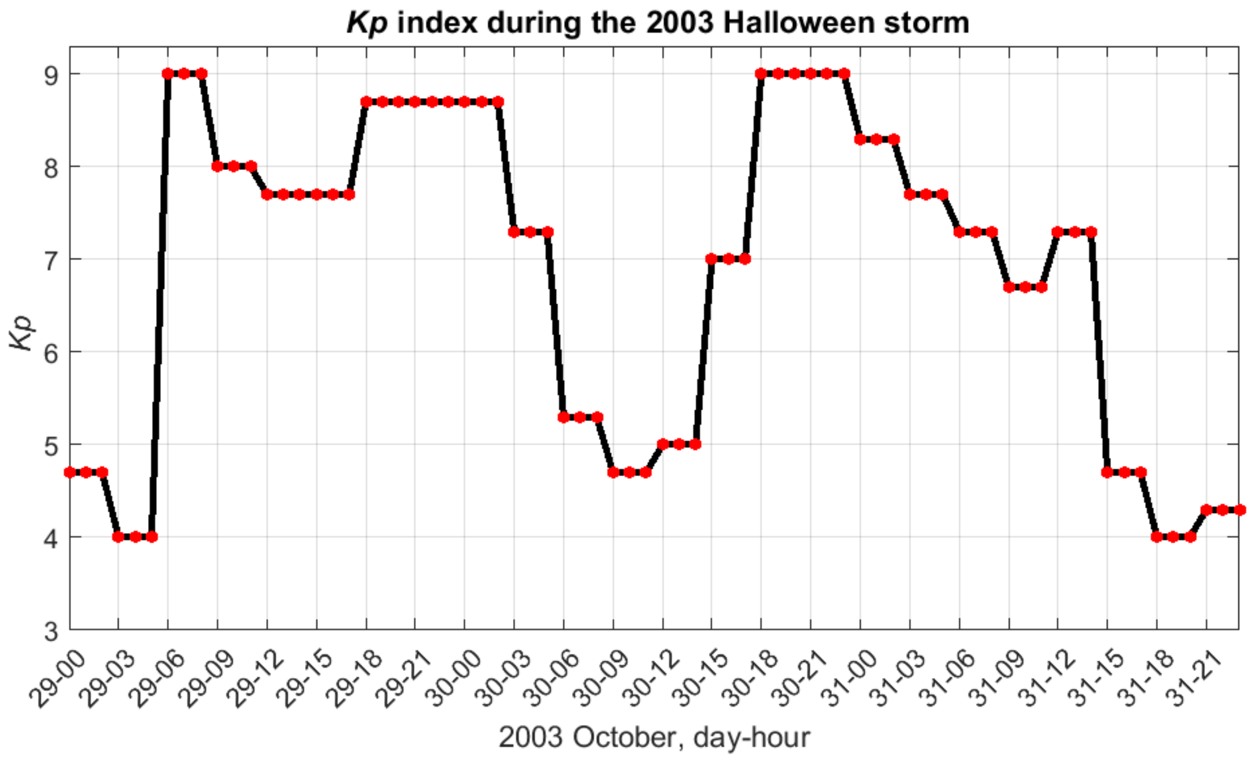

Supplementary Figure 4: *Kp* index during the October 2003 Halloween storm.

References

1. Ozeke, L. G., Mann, I. R., Murphy, K. R., Jonathan Rae, I. & Milling, D. K. Analytic expressions for ULF wave radiation belt radial diffusion coefficients. *Journal of Geophysical Research: Space Physics* **119**, 1587-1605 (2014).

2. Ozeke, L. G. *et al*. ULF wave derived radiation belt radial diffusion coefficients. *Journal of Geophysical Research: Space Physics* **117**, A04222 (2012).

3. Mann, I. *et al*. The upgraded CARISMA magnetometer array in the THEMIS era. *Space Science Reviews* **141**, 413-451 (2008).

4. Orlova, K., Shprits, Y. & Spasojevic, M. New global loss model of energetic and relativistic electrons based on Van Allen Probes measurements. *Journal of Geophysical Research: Space Physics* **121**, 1308-1314 (2016).

5. Gu, X., Shprits, Y. Y. & Ni, B. Parameterized lifetime of radiation belt electrons interacting with lower-band and upper-band oblique chorus waves. *Geophys. Res. Lett.* **39** (2012).

6. Carpenter, D. & Anderson, R. An ISEE/whistler model of equatorial electron density in the magnetosphere. *Journal of Geophysical Research: Space Physics* **97**, 1097-1108 (1992).
